# Relational reasoning in wild bumblebees revisited: the role of distance

**DOI:** 10.1038/s41598-023-49840-5

**Published:** 2023-12-15

**Authors:** Gema Martin-Ordas

**Affiliations:** Division of Psychology, University of Stirling, Stirling, UK

**Keywords:** Evolution, Psychology

## Abstract

In reasoning tasks, non-human animals attend more to relational than to object similarity. It is precisely this focus on relational similarity that has been argued to explain the reasoning gap between humans and other animals. Work with humans has revealed that objects placed near each other are represented to be more similar than objects placed farther apart. Will distance between objects also affect non-human animals’ abilities to represent and reason about objects? To test this, wild bumblebees were presented with a spatial reasoning task (with competing object matches) in which the objects or features alone (colour, shape) were placed close together or far apart. Bumblebees *spontaneously* attended to *objects* over relations, but only when the objects were far apart. Features alone were not strong enough to drive object matching—suggesting that bumblebees bound colour and shape into their object representations. These findings question whether the ability to focus on and compare objects is what makes human abstract reasoning unique.

## Introduction

Knowing the location of food resources and how to reach them are fundamental abilities for foraging animal species. In this context, two general strategies can be used for remembering where to find food: *object features* and *location* cues^1^. Whereas the former refers to individuals relying on features (e.g., colours, shapes) of the objects near a place of interest, the latter refers to individuals’ preferences for encoding and representing the location of an object *in relation to* another object^2,3^. In this regard, organisms can use two main strategies to represent spatial relationships: (1) *egocentric* strategies, which involve representing the location of an object relative to the speaker (e.g., the car is on my right); and (2) *object-centred* (i.e., allocentric) strategies, which involve representing locations relative to a landmark (e.g., the car is in front of the house). Using spatial relational paradigms, research has shown that there is a shared bias towards allocentric encoding of spatial relationships with humans and great apes’ common ancestor^2,4^. A recent study also using a relational paradigm revealed that invertebrates, in this case wild-caught bumblebees, likewise show this bias towards the use of allocentric strategies^5^.

When remembering the location of food, do non-human animals (henceforth animals) show a preference for the use of one type of cue (e.g., *location*) over the other (e.g., *object features*)? Numerous studies show that a wide range of vertebrate species can use both types of cues. For example, toads, pigeons, dogs, or lizards favour *location* cues^6–9^ and other species like chicks and goldfish prefer *object feature* cues^10,11^. Evidence also shows that invertebrates (e.g., insects) can learn to use *location* and *object features* when retrieving food sources^12,13^.

The findings just described are also significant in the context of relational reasoning, in general, and spatial mapping abilities, in particular. This is because understanding the relations of the properties of different objects in relation to each other—rather than the properties of the objects individually- is fundamental for recognizing relational similarity^2,4^. The capacity to perceive relational similarity (i.e., ability to, for example, align spatial relations across different sets of items) as different from object similarity (i.e., matching objects that look alike) impacts reasoning and is often considered a hallmark of human cognition. For instance, when asked the question “duck is to duckling as tiger to?” children answer “duckling” (*object* similarity) rather than “cub” (*relational* similarity). That is, children show a preference for object rather than relational similarity^4^. In contrast, animals (including invertebrates) favour relational similarity^14–17^. Why is this the case if animals can use both *object features* and *location* cues when finding food resources? Answering this question is critical because the ability to attend to objects rather than relations has been used to explain the gap in relational reasoning between humans and other animals^18^.

In research with adult humans, spatial distance between objects has been demonstrated to influence how adults represent and categorize objects (and their features). For example, when an apple and an orange are presented close together, adults perceive them as being more similar than when presented far apart^19,20^. Likewise, adults find it more difficult to decide which of two objects they prefer when the objects are presented close together compared to when they are not^21,22^. These results suggest that stimuli placed near each other are perceived to be more similar than stimuli placed farther apart. Closeness, thus, facilitates representing objects as being part of a same category^19,23^.

Here I investigated whether object distance affects wild-caught bumblebees’ abilities to recognize relational and object similarities in a spatial mapping task. For this purpose, a spatial relational task developed by Christie et al.^14^ and previously adapted for use with bumblebees^5^ was used. Specifically, I examined wild-caught bumblebees’ *spontaneous* preferences for *relational* and *object* similarity when the objects were placed close together compared to when they were placed farther apart (Experiments 1–3). It is expected that, like in previous studies^17^, bees show a preference for spatial matches compared to concrete objects when objects are placed close together. However, it is expected that bees show a preference for object matching when objects are placed far apart. This is because distance could facilitate identifying the features of the objects and, therefore, match them to the previously experienced ones^20^. Additionally, I examined the role that colour (Experiment 2) and shape (Experiment 3) play on bees’ relational abilities. This is important as it will shed light on what object features are relevant for bees to identify an object and whether the binding of these features—shape and colour- into the object representation is required for relational reasoning^25^. It is expected that if the object features are stored in the object representation, then these features independently will be available for deciding whether an object is one that has been encountered before (i.e., object matching).

## Experiment 1: object similarity vs spatial similarity

**Figure 1:**
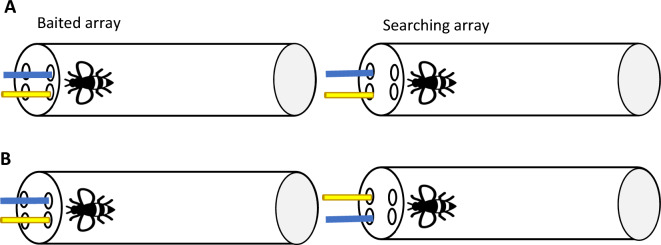
Bird’s-eye view of the experimental setup for *Near spatial reasoning* condition of Experiments 1–3. Bees experienced two objects (only one of them was dipped in sucrose) on the top array. Bees’ task was to search among the objects on the bottom array. Panel (**A**) depicts a control trial (spatial and object similarity match) and (**B**) an experimental trial (spatial match competes with object similarity match). The experimental setup of Experiments 2 and 3 was the same except for the objects used. In Experiment 2, a blue strip and a yellow paper stick were presented in the Baited Array and a yellow strip and a blue paper stick were presented in the Searching array. In Experiment 3, a yellow strip and a yellow paper stick were presented in the Baited Array and a blue strip and a blue paper stick were presented in the Searching array.

**Figure 2:**
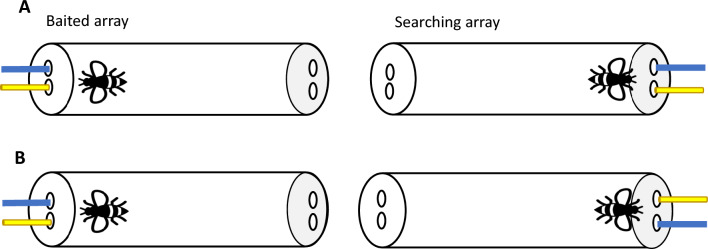
Bird’s-eye view of the experimental setup for *Distant spatial reasoning* condition of Experiments 1–3. Bees experienced two objects (only one of them was dipped in sucrose) in the Baited array. Bees’ task was to search among the objects on the Searching array. Panel (**A**) depicts a control trial (spatial and object similarity match) and (**B**) an experimental trial (spatial match competes with object similarity match). The experimental setup of Experiments 2 and 3 was the same except for the objects used. In Experiment 2, a blue strip and a yellow paper stick were presented in the Baited Array and a yellow strip and a blue paper stick were presented in the Searching array. In Experiment 3, a yellow strip and a yellow paper stick were presented in the Baited Array and a blue strip and a blue paper stick were presented in the Searching array.

Bumblebees experienced two sets (Baited and Searching arrays) of different objects (i.e., a blue strip and a yellow paper stick)^5,17^. First, they were presented with two objects in the Baited array—only one them was dipped in sucrose. Bees’ task was to find the corresponding object in the Searching array (see Figs. 1, 2). There were two conditions: *Near spatial reasoning* and *Distant spatial reasoning* (henceforth *Near* and *Distant,* respectively). In the *Near* condition, the Baited and Searching arrays were spatially aligned: top right object- > bottom right object, top left object- > bottom left object (Fig. 1). In the *Distant* condition, the two sets of objects were placed in a straight line on each side of the apparatus. In both conditions, there were two types of trials (Fig. 2). (a) Experimental trials: the objects in the Baited array were distributed so that the objects in the Searching array competed with the use of bees’ preferred spatial relational strategy (i.e., allocentric strategy)^5^; (b) Control trials: the objects in the Baited array were distributed so that the objects in the Searching array would match the use of the allocentric strategy (Fig. 1). It was predicted that if the distance between sets of objects does not affect bees’ spontaneous mapping strategies, bees would search top- > bottom in the *Near* condition and follow an allocentric strategy in the *Distant* condition—as found in a previous study^5^. However, if distance between sets of objects has an effect of bees’ spontaneous mapping strategies, bees would establish a spatial correspondence between the Baited and Searching arrays in the *Near* condition and an object correspondence in the *Distant* condition.

### Subjects

The data was collected between July and August 2022 in Stirlingshire (UK). Thirty-one bees were captured but one bee did not engage with the task. The final sample was integrated by 30 bees of the following species: *Bombus pascuorum* (n = 9), *Bombus terrestris complex* (n = 19), *Bombus hypnorum* (n = 1) and *Bombus monticola* (n = 1). Sex was visually identified (females = 26; males = 2 and 2 could not be clearly identified). No queens were tested in either of the 3 Experiments. This experiment as well as Experiments 2 and 3 received ethical approval from the University of Stirling’s Ethics Committee (Project name: Cognition in wild bees). All methods were performed in accordance with the relevant guidelines and regulations.

### Apparatus

Two types of tubes were used. For the *Near* task, a transparent plastic tube (14 × 3.5 cm) with two sets of 2 holes at the transparent end of the tube—one set of holes on top of the other- was used. The distance between the holes was 1 cm and the distance between the upper set of holes and the bottom one was 4 mm. For the *Distant* task, a transparent plastic tube (14 × 3.5 cm) with 2 holes at one end (transparent end) and 2 at the other end (lid end: light grey colour) was used. The distance between the holes—for transparent and lid ends- was equal (1 cm). In both tubes, the holes were used to introduce the stimuli (see Figs. 1&2). Blue strips of paper (3 × 0.2 cm) and yellow paper lollipop sticks (3 × 0.32 cm) were used as stimuli. Depending on the task, two stimuli were introduced through the transparent end of the tube or top set of holes (Baited array) and two through lid end of the tube or bottom set of holes (Searching array). The stimuli were fixed in playdoh to introduce them simultaneously in the tube.

### Procedure

Experiments were always conducted in the morning (7:30–10 am). Subjects were left in the tube on average for 1 h prior to testing to allow them to habituate to the tubes and become motivated to forage^26^. Bees were caught directly from flowers by using the testing tubes in which the experiments were conducted. This minimized the manipulation of the bees. Bees were captured throughout 15 days and an average of 2 bees per day were caught. After the experiment was over and before releasing the bees, they were individually marked. Posca markers were used for this purpose. As in Martin-Ordas^17^, bees were tested in the field on a T-shaped platform. On one of the sides of the platform 5 holes were drilled (Figure S1 in Supplementary Materials. The experimenter (E) sat in front of the platform and the tubes were inserted through the holes so that the transparent end always faced the E. To have the same environmental elements for all bees, playdoh containers were placed on both sides of the tube.

There were two conditions—*Near spatial reasoning* and *Distant spatial reasoning*- and bees only experienced one of them (Figs. 1, 2). The general procedure for both conditions was similar. First, subjects were presented with the Baited stimuli. Only one of the stimuli—left (e.g., yellow lollipop) or right (e.g., blue strip; Experimenter’s perspective, E)- was dipped in 50% (w/w) sucrose. Bees were allowed to explore both stimuli. Once the bee made contact with the stimulus dipped in sucrose—either by using its antennae or proboscis- it was given (on average) 5–6 s to drink the solution. Then, the Baited stimuli were removed, and E introduced the Searching stimuli below the Baited array (*Near* condition) or through the lid end (*Distant* condition) when the bees were not near the stimuli (i.e., 1.5–2 cm away from the stimuli). These strips were dipped in water. A choice was considered when the bees touched one of the strips of the Searching array with the antennae or proboscis^26^.

Each bee received a total of 12 trials—8 experimental trials and 4 control trials- either in the *Near* condition or in the *Distant* condition. In 6 of these trials, each bee experienced the yellow lollipop as the baited stimulus and in the other 6 trials, the blue strip as the baited stimulus. Which stimulus was rewarded and its location was counterbalanced across trials. The inter-trial-intervals were approximately 2 min for each bee and during this time, subjects were allowed to freely move in the tube. In the experimental trials, the stimuli in the Baited and Searching array followed an egocentric configuration; that is, the Searching stimuli maintained the same position relative to bees’ body axis as in the Baited array and, consequently, object matches competed with bees’ preferred spatial relational rule (i.e., allocentric searches). In the control trials, the stimuli followed an allocentric configuration—with the Searching stimuli maintaining the same position relative to a salient landmark (e.g., experimenter, other objects) as in the Baited array. Therefore, objects and spatial relational rule matched. The order in which bees received the experimental and control trials was randomized. New stimuli were used for each trial. Importantly, bees did not receive any training prior to these trials and their choices were not rewarded.

### Analyses

Data were analysed using R version 03.0 + 386 using a binomial general linear mixed model (GLMM)^27^. The dependent variable was whether bees’ choice in the Searching array of the experimental trials was correct (coded 1) or incorrect (coded 0), the independent variable was condition as a categorical variable and a random factor was the individual bees. A second model (see Supplementary Materials) was run including bee’s choice as dependent variable, condition as independent variable and individual bees, trial number and species as random factors. A comparison of bees’ performance in first vs last trial was included in Supplementary Materials. This was done to examine whether motivation had an effect in bees’ choices given that the stimuli in the Searching array were not rewarded. In the *Near* and *Distant* conditions, spatial matches were considered as correct. Wilcoxon tests were used to analyse if bees showed a preference for matching object or location and whether their performance in each condition was significantly above chance. Binomial tests were used to examine first trial performance. P-values below 0.050 were considered to provide evidence for significant differences.

## Results and discussion

**Figure 3:**
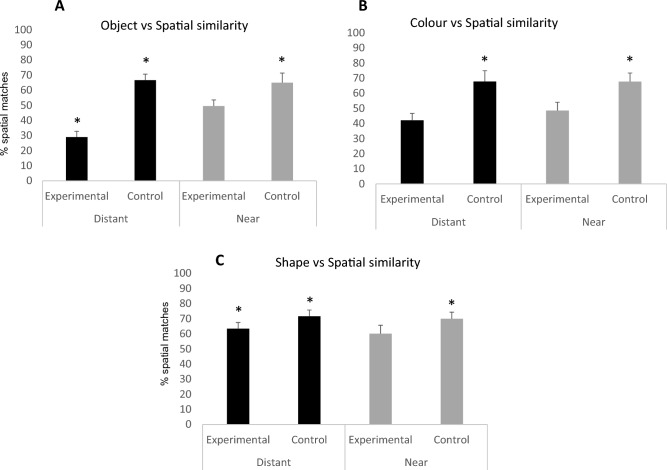
(**A**), (**B**) and (**C**) represents the percentage of spatial matches in the experimental and control trials of Experiment 1 (Object similarity vs Spatial Similarity), Experiment 2 (Colour similarity vs Spatial Similarity) and Experiment 3 (Shape similarity vs Spatial Similarity), respectively. The percentage of spatial matches was calculated out of the 12 trials performed for each bee. The individual percentages were then used to calculate the group mean. The asterisk indicates the conditions in which bees performed significantly above chance. Note that bees matched the objects significantly above chance in the Distant condition of Experiment 1. The bars represent the SEM.

There was a significant effect of condition in bees’ performance (estimate *SD* = − 0.870,* z* = − 3.20, *P* = 0.001, 95% CI = 0.246 to 0.714; Fig. 3A). When analysing each condition individually, it was found that in the *Distant* condition, bees focused on object matches more than on spatial matches (Wilcoxon test: *W* = 3, *P* = 0.002) and they did so significantly above chance (Wilcoxon test: *W* = 102, *P* = 0.002). Subjects did not show a preference for either strategy in the *Near* condition (Wilcoxon test: *W* = 31, *P* = 0.891; see Table 1 in Supplemental Materials (SM) for individual performances). In the control trials, subjects chose the correct item significantly above chance in the *Distant* (*W* = 45, *P* = 0.005) and *Near* (*W* = 48, *P* = 0.036) conditions. Bees were also significantly above chance at object matching in the first trial of the *Distant* condition (Binomial test, *P* = 0.021), but they did not exhibit a clear strategy in the first trial of the *Near* condition (Binomial test, *P* = 0.508).

As predicted, bees showed a preference for object matches in the *Distant* condition*,* but not in the *Near* condition. However, their responses in the *Near* condition also suggests that the presence of object matches was affecting bees’ searches. Individual performance indicated that whereas 33% of the subjects frequently used a relational strategy, 40% relied on an object matching strategy. Thus, bees exhibited both strategies in the *Near* condition. Previous studies have shown that infant humans and non-human primates can identify objects according to visual features such as shape or colour^29,30^. Importantly, bees have also been shown to use flower traits, such as shape or colour, to locate food sources^31^. Although different studies have demonstrated that some pollinators rely strongly on colour to make their foraging decisions^32–34^. Next, I examined the role that colour (Experiment 2) and shape (Experiment 3) plays in bees’ abilities in a relational task where the sets of objects are placed close together or far apart. These experiments will reveal whether a single feature of an object is enough to drive bees’ object match responses.

## Experiment 2: colour similarity vs spatial similarity

Similar to Experiment 1, bees were presented with Baited and Searching arrays in two conditions—*Near* or *Distant*. However, now in the experimental trials the objects in the Searching array were different (i.e., a yellow strip and a blue paper stick) from the objects in the Baited array (i.e., a blue strip and a yellow paper stick). Thus, in these trials the “colour” matches competed with the spatial relational rule (i.e., allocentric searches). It is predicted that if subjects were to focus on feature over relational matches, they would do so in the *Distant* condition but not in the *Near* condition. Consequently, a different performance in the *Near* compared to the *Distant* condition is expected.

### Subjects

Data was collected between June and August 2022 in Stirlingshire (UK). A total of 35 bees was captured, however 5 did not engage with the task and 1 failed to complete more than 4 trials and, therefore, was not included in the analyses. The final sample was integrated by 29 bees of the following species: *Bombus pascuorum* (n = 9), *Bombus terrestris complex* (n = 15), *Bombus hortorum* (n = 4), *Bombus hypnorum* (n = 1) and *Bombus monticola* (n = 1). Sex was visually identified (females = 26; males = 1 and 2 could not be clearly identified).

### Apparatus

The same materials as in Experiment 1 were used. Yellow strips of paper (3 × 0.2 cm) and blue paper lollipop sticks (3 × 0.32 cm) were also used as stimuli.

### Procedure

The same procedure as in Experiment 1 was followed. Bees were captured throughout 11 days and an average of 3 bees per day were caught. In the experimental trials, the stimuli in the Baited and Searching array followed an egocentric configuration (see Figs. 1&2). Therefore, colour matches competed with an allocentric search. The control trials were conducted as in Experiment 1.

### Analyses

The same analyses as in Experiment 1 were conducted.

## Results and discussion

There was no effect of condition in bees’ performance (estimate *SD* = − 0.226,* z* = − 0.824, *P* = 0.410, 95% CI = 0.465 to 1.37; Fig. 3B). Focusing on the experimental trials for each condition, bees did not show a preference for colour or spatial matching in either condition (*Distant*: Wilcoxon test: *W* = 28.5, *P* = 0.123; *Near*: Wilcoxon test: *W* = 37, *P* = 0.568; see Table 2 in SM for individual performances). In the control trials, when objects and spatial relational rule matched, subjects chose the correct item significantly above chance in both conditions (*Distant*: *W* = 74.5, *P* = 0.035; *Near*:* W* = 50.5, *P* = 0.019). Bees did not show a clear strategy in the first trial of the *Distant* condition (Binomial test, *P* = 1) nor of the *Near* condition (Binomial test, *P* = 0.424).

These results suggest that bees did not favour a feature (colour) matching nor a spatial matching strategy in either condition. This is similar to what research with infants has shown. The use of colour to identify objects emerges later than the use of shape—by 9 months infants bind shape to objects and by 12 they do so with colour^24^. In contrast, non-human primate research has shown that, in the context of food resources, primates use colour information more reliably than shape to identify items^29,35^. Note, though, that individual performance in the *Distant* condition showed that 71% of the subjects largely used a colour matching strategy. Experiment 3 examined whether bees’ mapping strategies could be driven only by the shape of the objects.

## Experiment 3: shape similarity vs spatial similarity

As in the previous Experiments, bees experienced a *Near* or a *Distant* condition. In the experimental trials, the objects in the Searching array were different (i.e., a blue strip and a blue paper stick) from the objects in the Baited array (i.e., a yellow strip and a yellow paper stick). Thus, in these trials the “shape” matches competed with the allocentric searches. It was expected that, like in other animal species, bees would struggle to use the shape feature and, therefore, relied on a spatial rather than feature matching strategy. However, if shape alone triggered object match strategies, bees would only do so in the *Distant* condition.

### Subjects

Data was collected in August 2022 in Stirlingshire (UK). Thirty-one bees were caught but one did not engage with the task. Thus, the final sample was integrated by 30 bees of the following species: *Bombus pascuorum* (n = 12), *Bombus terrestris complex* (n = 12) and *Bombus hortorum* (n = 6). Sex was visually identified (females = 24; males = 3 and 3 bees could not be clearly identified).

### Apparatus

The same materials as in Experiment 2 were used. Note that the paper strips were flat and rectangular, and the paper sticks were rounded.

### Procedure

The same procedure as in Experiment 2 was followed, with the only difference that now in the experimental trials, shape matches competed with the allocentric searches. Bees were captured throughout 6 days and an average of 5 bees per day were caught.

### Analyses

The same analyses as in the previous Experiments were conducted.

## Results and discussion

There was no effect of condition in bees’ performance (estimate *SD* = 0.122,* z* = 0.422, *P* = 0.673, 95% CI = 0.642 to 1.99; Fig. 3C). When selecting an item in the *Distant* condition, bees showed a preference for using a strategy based on spatial matching rather than feature (i.e., shape) matching (Wilcoxon test: *W* = 99.5, *P* = 0.003) and they did so significantly above chance (Wilcoxon test: *W* = 102, *P* = 0.003). As before, bees did not show a clear strategy in the *Near* condition (Wilcoxon test: *W* = 78.5, *P* = 0.104; see Table 3 in SM for individual performances). In the control trials, when object and location matched, subjects chose the correct item significantly above chance in both conditions (*Distant*: *W* = 66, P = 0.002; *Near*:* W* = 55, *P* = 0.004). Bees did not show a clear strategy in the first trial of the *Distant* (Binomial test, *P* = 0.344) nor *Near* condition (Binomial test, *P* = 0.727).

Overall, these results indicate that in the *Distant* condition bees consistently relied on a spatial matching strategy. As before, bees did not show a strong preference for the use of spatial or feature matching strategy in the *Near* condition. Thus, in the present paradigm, shape alone was not salient enough to drive bees’ object matching responses. This is similar to what it has been found in studies with non-human primates: shape was only taken into account when subjects were provided with previous experience with this feature^29,35^. Thus, it is possible that the nature of the paradigm used here—i.e., lack of previous experience with the two shapes and/or responses not being rewarded- affected bees’ performance. One would expect that if bees had been given this experience, they might have been able to consistently exploit shape matches.

## General discussion

Wild bumblebees consistently attended to objects matches when the sets of objects were placed apart compared to when they were placed close together. Examining what features bees used to identify and match objects revealed that neither colour alone nor shape alone drove bees’ object matching strategies. Moreover, shape largely prompted a spatial matching strategy.

The results presented here confirm previous findings by showing that bees are able of *both* relational and object similarity^17^ and extend them by demonstrating that distance between sets of objects can determine bees’ mapping strategies. It is possible that when the sets of objects were in close distance, bees represented them as being “similar”—which could explain why individuals did not show a clear preference for object or spatial matching. After all, in nature objects that are close to each other often also are similar to each other and belong to the same category^19,23^. For instance, flowers of the same species are often close to other flowers of the same species. In contrast, placing the sets of objects farther apart could have facilitated bees identifying similarities and differences between the objects—which might explain why bees displayed an object matching strategy.

The current studies also shed light on what features bees use to identify and match objects in small-scale relational mapping paradigms. Individual performance in Experiment 2 showed that bees, to some extent, pay attention to colour to identify objects. However, it is difficult to establish a strong conclusion regarding bees’ failure to use shape. It is conceivable that bees might use an extremely salient shape difference for object identification or that the shapes used in the current study were not noticeable enough for them. Importantly, although shape and colour alone did not reliably prompt an object mapping strategy, it is plausible that bees bound shape and colour into their representation of the objects. This is because in Experiment 1 bees matched objects significantly above chance in the *Distant* condition. Moreover, when bees did not rely on objects’ individual features, they consistently switched to a spatial matching strategy (e.g., Experiment 3). This is a fascinating result not only because it demonstrates that they encoded the spatial information of the objects but also because it shows that bees use these types of strategies in a flexible manner.

Even though previous work has demonstrated that bees can learn to abstract relational representations (i.e., “sameness”) in the context of colours, smells, sizes and quantities^35–37^, they do not favour object similarity in relational reasoning tasks^17^. Thus, these results provide some of the first evidence that insects, in particular bumblebees, *spontaneously* favour *object* over spatial matching in mapping tasks—although it only occurs when objects are placed far apart. This is in contrast to humans. For example, when two matching choices are available, children strongly prefer object similarity over relational matches^15,16, 39^—and this seems to be independent of the distance between the objects. It has been argued that sensitivity to object similarity can be helpful in learning to identify relations. The saliency of perceptual similitude is what invites individuals to compare objects and this comparison is what facilitates discovering shared relational structure between objects. For example, comparing the physical similarity between bicycles and tricycles would allow to establish that skateboards are also vehicles—even if they are perceptually different from other items in the same category^15,39^. The finding that bees show a proclivity for object matching in particular contexts suggests that this initial invitation to compare may not be uniquely human—questioning whether the effects of object comparisons over time is what contributes to the human advantage in abstract reasoning.

Previous research has shown that bees are sensitive to shape and colour of the flowers^31^. Even though the presence of feature matches affected bees’ performance in the current experiments, bees did not reliably match colour or shape. It is possible that the nature of the paradigm used here—i.e., number of trials and/or responses not being rewarded- affected bees’ performance. One would expect that if they had been given more trials and their searches had been rewarded, they might have been able to consistently exploit features matches. A surprising finding was that when presented with shape alone, bees displayed a spatial matching strategy. As mentioned before, it is possible that the shape feature in Experiment 3 was not salient enough for the bees. Thus, future studies should address this issue by presenting bees with shapes that are noticeable enough for them in the context of small-scale spatial tasks. The small sample of males included in these studies did not allow to explore sex differences in bees’ reasoning strategies. While it is true that male and female bumblebees perform different ecological roles, finding flowers using either object or location matching strategies would be equally beneficial for males and females. This leaves open the possibility that the different roles played by males and females do not necessarily affect their cognitive abilities—as previous research has already shown in the context of learning^39,40^. Future research should investigate this possibility. Finally, the paradigms in which these cognitive abilities were examined were intended for testing wild bees. Thus, certain factors such individual experience could not be controlled for. Future research with laboratory bees could help to shed light on whether individual learning experience plays an important role in reasoning paradigms.

To conclude, bumblebees displayed a flexible use of spatial and object matching strategies—suggesting that the context and object features play a critical role on which reasoning strategies bees use. Studies like the ones presented here indicate that research with social insects is very useful to investigate the evolution of cognition, in general, and what factors are at play in cognition in insects, in particular.

### Ethical approval

The experiments received ethical approval from the University of Stirling’s Ethics Committee (Project name: Cognition in wild bees; Project number: 4041).

### Supplementary Information

Supplementary Information.

## Data Availability

Data are included in the Supplemental Information.
