# Supplementary material for: Relational reasoning in wild bumblebees revisited: the role of distance
Source: Sci Rep. 2023 Dec 15;13:22311. doi: 10.1038/s41598-023-49840-5 (PMC10724225; doi:10.1038/s41598-023-49840-5)
Supplement: Supplementary file 1 — Supplementary Information. [file 41598_2023_49840_MOESM1_ESM.docx]

**Supplementary Materials**

**Figure S1**. T-shaped platform and experimental setup used for the *Distant* condition experiments. Tubes are placed parallel to the ground and inserted through a hole on the T-shaped platform so they do not move during the testing. Two air holes were drilled into the tube. The setup for the *Near* condition is the same as for *Distant* condition with the only difference that the Searching array is right below the Baiting array.

**Experiment 1: Object similarity vs spatial similarity**

**Table 1.** Individual performance in Experiment 1: Object similarity vs spatial similarity

| Subject | Condition | Location | Object | Species |
| --- | --- | --- | --- | --- |
| 146 | *Distant* | 12.5 | **87.5** | *B. pascuorum* |
| 147 | *Distant* | 50 | 50 | *B. terrestris complex* |
| 150 | *Distant* | 25 | **75** | *B. pascuorum* |
| 151 | *Distant* | 25 | **75** | *B. pascuorum* |
| 155 | *Distant* | 37.5 | **62.5** | *B. terrestris complex* |
| 156 | *Distant* | 37.5 | **62.5** | *B. terrestris complex* |
| 159 | *Distant* | **62.5** | 37.5 | *B. terrestris complex* |
| 160 | *Distant* | 12.5 | **87.5** | *B. terrestris complex* |
| 161 | *Distant* | 25 | **75** | *B. terrestris complex* |
| 162 | *Distant* | 12.5 | **87.5** | *B. terrestris complex* |
| 166 | *Distant* | 25 | **75** | *B. pascuorum* |
| 167 | *Distant* | 25 | **75** | *B. pascuorum* |
| 168 | *Distant* | 37.5 | **62.5** | *B. hypnorum* |
| 169 | *Distant* | 12.5 | **87.5** | *B. terrestris complex* |
| 172 | *Distant* | 37.5 | **62.5** | *B. pascuorum* |
| 173 | *Near* | **62.5** | 37.5 | *B. terrestris complex* |
| 176 | *Near* | 25 | **75** | *B. terrestris complex* |
| 181 | *Near* | 50 | 50 | *B. terrestris complex* |
| 184 | *Near* | 42.8 | **57.14** | *B. monticola* |
| 188 | *Near* | **62.5** | 37.5 | *B. terrestris complex* |
| 189 | *Near* | 37.5 | **62.5** | *B. terrestris complex* |
| 195 | *Near* | 50 | 50 | *B. terrestris complex* |
| 196 | *Near* | **87.5** | 12.5 | *B. terrestris complex* |
| 201 | *Near* | **62.5** | 37.5 | *B. terrestris complex* |
| 204 | *Near* | 50 | 50 | *B. terrestris complex* |
| 206 | *Near* | 25 | **75** | *B. terrestris complex* |
| 207 | *Near* | 37.5 | **62.5** | *B. pascuorum* |
| 217 | *Near* | **62.5** | 37.5 | *B. pascuorum* |
| 222 | *Near* | 50 | 50 | *B. pascuorum* |
| 225 | *Near* | 37.5 | **62.5** | *B. terrestris complex* |

Table 1 represents the individual performance in Experiment 1—both *Distant* and *Near* conditions. Whereas in the *Distant* condition 87% of the subjects consistently used an object matching strategy, in the *Near* condition 40% of the subjects did so and 33% tended to rely on a spatial mapping strategy. These results suggest that some bees were sensitive to the competing object matches in the *Near* condition.

When the model was run including condition as fixed factors and individuals, trial number and species as random factors, the results showed that condition had an effect on subjects’ performance: condition (estimate *SD* = -0.870, *z* = -3.20, *P* = 0.001, 95% CI = 0.246 to 0.714). There was no difference in performance between first and last trial (estimate *SD* = -0.888, *z* = -1.553, *P* = 0.120, 95% CI = 0.134 to 1.26), thus lack of motivation did not affect bees’ choices.

**Experiment 2: Colour similarity vs spatial similarity**

**Table 2.** Individual performance in Experiment 2: Colour similarity vs spatial similarity

| Subject | Condition | Location | Colour | Species |
| --- | --- | --- | --- | --- |
| 3 | *Distant* | **75** | 25 | *B. terrestris complex* |
| 4 | *Distant* | 37.5 | **62.5** | *B. hypnorum* |
| 215 | *Distant* | 37.5 | **62.5** | *B. terrestris complex* |
| 219 | *Distant* | **62.5** | 37.5 | *B. terrestris complex* |
| 223 | *Distant* | **62.5** | 37.5 | *B. pascuorum* |
| 230 | *Distant* | 37.5 | **62.5** | *B. monticola* |
| 232 | *Distant* | 37.5 | **62.5** | *B. terrestris complex* |
| 236 | *Distant* | 37.5 | **62.5** | *B. terrestris complex* |
| 239 | *Distant* | **62.5** | 37.5 | *B. terrestris complex* |
| 243 | *Distant* | 37.5 | **62.5** | *B. terrestris complex* |
| 246 | *Distant* | 25 | **75** | *B. terrestris complex* |
| 253 | *Distant* | 37.5 | **62.5** | *B. hortorum* |
| 254 | *Distant* | 15 | **85** | *B. pascuorum* |
| 260 | *Distant* | 25 | **75** | *B. pascuorum* |
| 247 | *Near* | 37.5 | **62.5** | *B. pascuorum* |
| 248 | *Near* | **62.5** | 37.5 | *B. pascuorum* |
| 231 | *Near* | 37.5 | **62.5** | *B. terrestris complex* |
| 234 | *Near* | 50 | 50 | *B. terrestris complex* |
| 235 | *Near* | **62.5** | 37.5 | *B. terrestris complex* |
| 255 | *Near* | 25 | **75** | *B. hortorum* |
| 252 | *Near* | **66.66** | 33.33 | *B. pascuorum* |
| 259 | *Near* | **62.5** | 37.5 | *B. pascuorum* |
| 264 | *Near* | **62.5** | 37.5 | *B. hortorum* |
| 267 | *Near* | 50 | 50 | *B. hortorum* |
| 268 | *Near* | **100** | 0 | *B. pascuorum* |
| 265 | *Near* | 25 | **75** | *B. pascuorum* |
| 271 | *Near* | 25 | **75** | *B. terrestris complex* |
| 228 | *Near* | 37.5 | **62.5** | *B. terrestris complex* |
| 256 | *Near* | 25 | **75** | *B. terrestris complex* |

Table 2 represents the individual performance in Experiment 2—both *Distant* and *Near* conditions. Whereas in the *Distant* condition 71% of the subjects frequently used an colour matching strategy, in the *Near* condition 47% of the subjects did so and 40% tended to rely on a spatial mapping strategy. These results suggest that some bees were sensitive to the competing colour matches in the *Distant* and *Near* conditions.

When the model was run including condition as fixed factors and individuals, trial number and species as random factors, the results showed that condition did not have a significant effect (estimate *SD* = -0.226, *z* = -0.824, *P* = 0.410, 95% CI = 0.465 to 1.37). There was no difference in performance between first and last trial (estimate *SD* = -0.678, *z* = -1.233, *P* = 0.217, 95% CI = 0.173 to 1.49), thus lack of motivation did not affect bees’ choices.

**Experiment 3: Shape similarity vs spatial similarity**

**Table 3.** Individual performance in Experiment 3: Shape similarity vs spatial similarity

| Subject | Condition | Location | Shape | Species |
| --- | --- | --- | --- | --- |
| 261 | *Distant* | **75** | 25 | *B. terrestris complex* |
| 262 | *Distant* | 42.85 | **57.15** | *B. terrestris complex* |
| 269 | *Distant* | **67** | 33 | *B. hortorum* |
| 270 | *Distant* | **87.5** | 12.5 | *B. pascuorum* |
| 272 | *Distant* | **62.5** | 37.5 | *B. terrestris complex* |
| 273 | *Distant* | **87.5** | 12.5 | *B. terrestris complex* |
| 276 | *Distant* | **62.5** | 37.5 | *B. hortorum* |
| 278 | *Distant* | **62.5** | 37.5 | *B. hortorum* |
| 279 | *Distant* | **62.5** | 37.5 | *B. terrestris complex* |
| 280 | *Distant* | **67** | 33 | *B. terrestris complex* |
| 282 | *Distant* | 37.5 | 62.5 | *B. hortorum* |
| 287 | *Distant* | **75** | 25 | *B. pascuorum* |
| 288 | *Distant* | **62.5** | 37.5 | *B. pascuorum* |
| 291 | *Distant* | 50 | 50 | *B. pascuorum* |
| 296 | *Distant* | **75** | 25 | *B. pascuorum* |
| 281 | *Near* | **62.5** | 37.5 | *B. terrestris complex* |
| 283 | *Near* | 12.5 | **87.5** | *B. pascuorum* |
| 284 | *Near* | **87.5** | 12.5 | *B. terrestris complex* |
| 285 | *Near* | **75** | 25 | *B. pascuorum* |
| 286 | *Near* | 37.5 | **62.5** | *B. terrestris complex* |
| 289 | *Near* | **57** | 43 | *B. pascuorum* |
| 290 | *Near* | **100** | 0 | *B. pascuorum* |
| 292 | *Near* | **62.5** | 37.5 | *B. pascuorum* |
| 293 | *Near* | **62.5** | 37.5 | *B. terrestris complex* |
| 294 | *Near* | 50 | 50 | *B. pascuorum* |
| 295 | *Near* | **62.5** | 37.5 | *B. terrestris complex* |
| 258 | *Near* | 33 | **77** | *B. terrestris complex* |
| 274 | *Near* | **75** | 25 | *B. hortorum* |
| 277 | *Near* | **62.5** | 37.5 | *B. pascuorum* |
| 275 | *Near* | **62.5** | 37.5 | *B. hortorum* |

Table 3 represents the individual performance in Experiment 3—both *Distant* and *Near* conditions. Whereas in the *Distant* condition 87% of the subjects frequently used a spatial matching strategy, in the *Near* condition 73% of the subjects did so and 20% tended to rely on a shape mapping strategy. These results suggest that some bees relied on spatial mapping in both conditions.

When the model was run including condition as fixed factors and individuals, trial number and species as random factors, the results showed that condition did not have a significant effect (estimate *SD* = 0.122, *z* = 0.422, *P* = 0.673, 95% CI = 0.642 to 1.99). There was no difference in performance between first and last trial (estimate *SD* = -0.194, *z* = -0.351, *P* = 0.725, 95% CI = 0.280 to 2.43), thus lack of motivation did not affect bees’ choices.

**Notes**

* Individual performance refers to the percentage of trials in which each bee used either a location matching strategy or an object (Experiment 1)/colour (Experiment 2)/shape (Experiment 3) strategy.
